# Supplementary material for: Advancing Archaeobotanical Methods: Morphometry, Bayesian Analysis and AMS Dating of Rose Prickles from Monteagudo Almunia, Spain (12th Century–Present)
Source: Plants (Basel). 2025 Dec 5;14(24):3709. doi: 10.3390/plants14243709 (PMC12736896; doi:10.3390/plants14243709)
Supplement: Supplementary file 1 [file plants-14-03709-s001.zip › plants-4001506-supplementary.pdf]

Supplementary Table S1. Prickle types proportions in the analyzed stem and leaf rachis fragments

Ninety-one stem fragments and seven leaf rachis fragments from sixteen *Rosa* taxa (species and varieties), and two stem fragments and three leaf rachis fragments *Rubus ulmifolius* were analyzed as modern reference materials. Furthermore four archaeological carbonized rose stem fragments from Oplontis (Italy) were studied.

	Taxa	Variety	T1 prickle (2.5 mm l x 3 mm b)	T2 prickle (5 mm l x 2 mm b)	T3 or TO, other prickle types	Total prickles per sample	Length of the sample (cm)
1.	Rosa x alba L.	Rosa x alba "Felicité Parmentier"	1	4	40	45	5
2.	Rosa x alba L.	Rosa x alba "Felicité Parmentier"	0	2	56	58	6
3.	Rosa x alba L.	Rosa x alba "Felicité Parmentier"	0	5	26	31	5.4
4.	Rosa x alba L.	Rosa x alba "Felicité Parmentier"	0	3	25	28	3.5
5.	Rosa x alba L.	Rosa x alba "York & Lancaster"	1	3	65	69	5.7
6.	Rosa x alba L.	Rosa x alba "York & Lancaster"	1	2	75	78	5
7.	Rosa x alba L.	Rosa x alba "York & Lancaster"	1	2	37	40	4.9
8.	Rosa x alba L.	Rosa x alba "York & Lancaster"	2	0	55	57	5.3
9.	Rosa x alba L.	Rosa x alba "York & Lancaster"	1	2	70	73	5.5
10.	Rosa x centifolia L.	Rosa x centifolia "Muscosa"	0	3	166	169	6.5
11.	Rosa x centifolia L.	Rosa x centifolia "Muscosa"	0	9	168	177	6.7

Taxa	Variety	T1 prickle (2.5 mm l x 3 mm b)	T2 prickle (5 mm l x 2 mm b)	T3 or TO, other prickle types	Total prickles per sample	Length of the sample (cm)
12. Rosa x centifolia L.	Rosa x centifolia "Muscosa"	1	10	144	155	6.8
13. Rosa gallica L.	Rosa gallica	0	0	20	20	4.5
14. Rosa gallica L.	Rosa gallica	0	0	19	19	4
15. Rosa gallica L.	Rosa gallica	0	0	13	13	5.4
16. Rosa gallica L.	Rosa gallica	0	0	19	19	4.8
17. Rosa x damascena Herrm.	Rosa x damascena "Kazanlik"	0	0	76	76	3.2
18. Rosa x damascena Herrm.	Rosa x damascena "Kazanlik"	0	2	116	118	5.7
19. Rosa x damascena Herrm.	Rosa x damascena "Kazanlik"	2	1	152	155	6.6
20. Rosa x damascena Herrm.	Rosa x damascena "Kazanlik"	2	3	142	147	6.2
21. Rosa sp. Tea	Rosa sp. Tea	0	0	3	3	5
22. Rosa sp. Tea	Rosa sp. Tea	0	0	6	6	6
23. Rosa sp. Tea	Rosa sp. Tea	0	0	4	4	5.9
24. Rosa foetida Herrm.	Rosa foetida "Mesones"	0	1	2	3	4.3
25. Rosa foetida Herrm.	Rosa foetida "Mesones"	0	2	2	4	5.5
26. Rosa foetida Herrm.	Rosa foetida "Mesones"	0	0	2	2	5.8
27. Rosa foetida Herrm.	Rosa foetida "Mesones"	0	1	3	4	6

Taxa	Variety	T1 prickle (2.5 mm l x 3 mm b)	T2 prickle (5 mm l x 2 mm b)	T3 or TO, other prickle types	Total prickles per sample	Length of the sample (cm)
28. Rosa foetida Herrm.	Rosa foetida "Mesones"	0	1	3	4	5
29. Rosa foetida Herrm.	Rosa foetida "Mesones"	0	1	2	3	5.7
30. Rosa foetida Herrm.	Rosa foetida "Mesones"	0	1	2	3	6
31. Rosa lutea var. persiana Lem. (R. foetida f. persiana (Lem.) Rehde)r	Rosa persiana	0	9	5	14	7.3
32. Rosa lutea var. persiana Lem. (R. foetida f. persiana (Lem.) Rehde)	Rosa persiana	0	5	0	5	6.7
33. Rosa lutea var. persiana Lem. (R. foetida f. persiana (Lem.) Rehde)	Rosa persiana	0	4	1	5	3.8
34. Rosa lutea var. persiana Lem. (R. foetida f. persiana (Lem.) Rehde)	Rosa persiana	0	4	14	18	6.8
35. Rosa gallica L.	Rosa gallica "Officinalis"	0	0	23	23	5.2
36. Rosa gallica L.	Rosa gallica "Officinalis"	0	1	12	13	5
37. Rosa gallica L.	Rosa gallica "Officinalis"	0	0	20	20	6.5
38. Rosa gallica L.	Rosa gallica "Officinalis"	0	0	11	11	5.7
39. Rosa gallica L.	Rosa gallica "Officinalis"	0	1	5	6	5.4
40. Rosa gallica L.	Rosa gallica "Officinalis"	0	1	10	11	6
41. Rosa gallica L.	Rosa gallica "Officinalis"	0	1	16	17	6
42. Rosa gallica L.	Rosa gallica "Officinalis"	0	1	19	20	5.2
43. Rosa gallica L.	Rosa gallica "Officinalis"	0	0	29	29	5.5

Taxa	Variety	T1 prickle (2.5 mm l x 3 mm b)	T2 prickle (5 mm l x 2 mm b)	T3 or TO, other prickle types	Total prickles per sample	Length of the sample (cm)
44. Rosa hemisphaerica Herrm.	Rosa hemisphaerica	1	3	72	76	6
45. Rosa hemisphaerica Herrm.	Rosa hemisphaerica	0	5	98	103	6.5
46. Rosa hemisphaerica Herrm.	Rosa hemisphaerica	0	2	63	65	7.5
47. Rosa bicolor Jacq.	Rosa bicolor	0	0	48	48	5
48. Rosa bicolor Jacq.	Rosa bicolor	0	0	48	48	5
49. Rosa moschata Herrm.	Rosa moschata "Umbrella" ST	1	0	6	7	6
50. Rosa moschata Herrm.	Rosa moschata "Umbrella" ST	0	0	1	1	6
51. Rosa moschata Herrm.	Rosa moschata "Umbrella" ST	3	0	4	7	6.8
52. Rosa moschata Herrm.	Rosa moschata "Umbrella" ST	0	0	6	6	6.3
53. Rosa moschata Herrm.	Rosa moschata "Umbrella" ST	1	0	1	2	7.4
54. Rosa moschata Herrm.	Rosa moschata "Umbrella" ST	0	0	3	3	6.8
55. Rosa moschata Herrm.	Rosa moschata "Umbrella" LR	1	0	2	3	5
56. Rosa moschata Herrm.	Rosa moschata "Umbrella" LR	1	0	2	3	5
57. Rosa moschata Herrm.	Rosa moschata "Umbrella" LR	2	0	2	4	3.3
58. Rosa rubiginosa L.	Rosa rubiginosa	0	0	8	8	5.9
59. Rosa rubiginosa L.	Rosa rubiginosa	0	0	2	2	3.9
60. Rosa rubiginosa L.	Rosa rubiginosa	0	0	8	8	4.6

Taxa	Variety	T1 prickle (2.5 mm l x 3 mm b)	T2 prickle (5 mm l x 2 mm b)	T3 or TO, other prickle types	Total prickles per sample	Length of the sample (cm)
61. Rosa rubiginosa L.	Rosa rubiginosa	0	0	3	3	3.4
62. Rosa rubiginosa L.	Rosa rubiginosa	0	2	13	15	6.2
63. Rosa rubiginosa L.	Rosa rubiginosa	0	3	14	17	5.8
64. Rosa sempervirens Savi	Rosa sempervirens Low Stem	0	0	4	4	6.6
65. Rosa sempervirens Savi	Rosa sempervirens Low Stem	0	0	2	2	6.9
66. Rosa sempervirens Savi	Rosa sempervirens Low Stem	0	0	3	3	6.8
67. Rosa sempervirens Savi	Rosa sempervirens Low Stem	0	0	3	3	7
68. Rosa sempervirens Savi	Rosa sempervirens Low Stem	0	0	4	4	7.5
69. Rosa sempervirens Savi	Rosa sempervirens Low Stem	0	0	3	3	7
70. Rosa sempervirens Savi	Rosa sempervirens Low Stem	0	0	5	5	6.6
71. Rosa sempervirens Savi	Rosa sempervirens Upper P Stem	1	0	6	7	6.6
72. Rosa sempervirens Savi	Rosa sempervirens Upper P Stem	1	0	9	10	7
73. Rosa sempervirens Savi	Rosa sempervirens Upper P Stem	1	0	5	6	5
74. Rosa sempervirens Savi	Rosa sempervirens Upper P Stem	2	0	6	8	6.7

Taxa	Variety	T1 prickle (2.5 mm l x 3 mm b)	T2 prickle (5 mm l x 2 mm b)	T3 or TO, other prickle types	Total prickles per sample	Length of the sample (cm)
75. Rosa sempervirens Savi	Rosa sempervirens Upper P Stem	0	0	7	7	7.2
76. Rosa sempervirens Savi	Rosa sempervirens Upper P Stem	0	0	8	8	6.6
77. Rosa x bifera (Poir.) Pers.	Rosa bifera	0	5	56	61	8
78. Rosa x bifera (Poir.) Pers.	Rosa bifera	0	2	40	42	4.2
79. Rosa x bifera (Poir.) Pers.	Rosa bifera	0	2	36	38	5.8
80. Rosa x bifera (Poir.) Pers.	Rosa bifera	0	4	56	60	7.6
81. Rosa x bifera (Poir.) Pers.	Rosa bifera	0	1	37	38	5.8
82. Rosa x bifera (Poir.) Pers.	Rosa bifera	0	4	45	49	8.5
83. Rosa x bifera (Poir.) Pers.	Rosa bifera	0	4	41	45	4.3
84. Rosa x bifera (Poir.) Pers.	Rosa bifera	0	2	35	37	8.2
85. Rosa canina L.	Rosa canina ST	0	0	4	4	5.2
86. Rosa canina L.	Rosa canina ST	0	0	3	3	4.9
87. Rosa canina L.	Rosa canina ST	0	0	4	4	5.9
88. Rosa canina L.	Rosa canina ST	0	0	4	4	6.3
89. Rosa canina L.	Rosa canina LE	6	0	2	8	4
90. Rosa canina L.	Rosa canina LE	3	0	1	4	3.5

Taxa	Variety	T1 prickle (2.5 mm l x 3 mm b)	T2 prickle (5 mm l x 2 mm b)	T3 or TO, other prickle types	Total prickles per sample	Length of the sample (cm)
91. Rosa canina L.	Rosa canina LE	2	0	2	4	6
92. Rosa canina L.	Rosa canina LE	6	0	1	7	6
93. Rosa agrestis Savi	Rosa agrestis ALM	1	0	10	11	8
94. Rosa agrestis Savi	Rosa agrestis ALM	0	1	11	12	9
95. Rosa agrestis Savi	Rosa agrestis ALM	0	1	16	17	11
96. Rosa agrestis Savi	Rosa agrestis MAIGM	2	0	8	10	6.5
97. Rosa agrestis Savi	Rosa agrestis MAIGM	4	0	6	10	9
98. Rosa agrestis Savi	Rosa agrestis MAIGM	1	1	14	16	12
1. Rubus ulmifolius Schott.	Rubus ulmifolius ST	0	0	4	4	7
2. Rubus ulmifolius Schott.	Rubus ulmifolius ST	0	0	6	6	6.1
3. Rubus ulmifolius Schott.	Rubus ulmifolius LE	0	0	8	8	5
4. Rubus ulmifolius Schott.	Rubus ulmifolius LE	0	0	9	9	5
5. Rubus ulmifolius Schott.	Rubus ulmifolius LE	0	0	10	10	5
1. Rosa oplontis	Rosa Oplontis A	0	1	0	1	5
2. Rosa oplontis	Rosa Oplontis B	2	0	0	2	4
3. Rosa oplontis	Rosa Oplontis C	1	0	0	1	3.5

Taxa	Variety	T1 prickle (2.5 mm l x 3 mm b)	T2 prickle (5 mm l x 2 mm b)	T3 or TO, other prickle types	Total prickles per sample	Length of the sample (cm)
4. Rosa oplontis	Rosa Oplontis D	1	0	9	10	10
